# High-Performance Self-powered Photodetectors Based on ZnO/ZnS Core-Shell Nanorod Arrays

**DOI:** 10.1186/s11671-016-1639-7

**Published:** 2016-09-22

**Authors:** Hailing Lin, Lin Wei, Cuncun Wu, Yanxue Chen, Shishen Yan, Liangmo Mei, Jun Jiao

**Affiliations:** 1 School of Physics and State Key Laboratory of Crystal Materials, Shandong University, Jinan, 250100 People’s Republic of China; 2 School of Microelectronics, Shandong University, Jinan, 250100 People’s Republic of China; 3 Department of Mechanical and Materials Engineering, Portland State University, P.O. Box 751, Portland, OR 97207-0751 USA

**Keywords:** ZnO/ZnS heterojunction, Nanorod arrays, Self-powered, Ultraviolet photodetectors

## Abstract

In recent years, there is an urgent demand for high-performance ultraviolet photodetectors with high photosensitivity, fast responsivity, and excellent spectral selectivity. In this letter, we report a self-powered photoelectrochemical cell-type UV detector using the ZnO/ZnS core-shell nanorod array as the active photoanode and deionized water as the electrolyte. This photodetector demonstrates an excellent spectral selectivity and a rapid photoresponse time of about 0.04 s. And the maximum responsivity is more than 0.056 (A/W) at 340 nm, which shows an improvement of 180 % compared to detectors based on the bare ZnO nanorods. This improved photoresponsivity can be understood from the step-like band energy alignment of the ZnO/ZnS interface, which will accelerate the separation of photoexcited electron-hole pairs and improve the efficiency of the photodetector. Considering its uncomplicated low-cost fabrication process, and environment-friendly feature, this self-powered device is a promising candidate for UV detector application.

## Background

Ultraviolet light has been widely used in secure space-to-space communication, chemical sensing, environmental monitoring, and optoelectronic circuits [1–3]. For most of these applications, it is very important to precisely measure and control UV light. There are typically two types of ultraviolet photodetectors (UV-PDs), e.g., photoconductor- [4] and photodiode-type [5] UV-PDs. So far, many wide bandgap semiconductors, such as ZnO [6], TiO_2_ [7], SnO_2_ [8], K_2_Nb_8_O_21_ [9], and ZnS [10, 11], have been explored for use in photoconductor-type UV-PDs. However, this type of UV-PDs normally involves the absorption and desorption of oxygen in the air, both of which are slow processes. Besides, this type of UV photoconductors typically requires an external bias as the driving force to prevent the recombination of photogenerated electron-hole pairs, which makes them difficult to operate in harsh environment. To address these problems, self-powered photodiode-type UV-PDs have been developed. Photodiode-type UV-PDs can convert the incident light into an electrical signal output with no need of an external power supply. And these photodiodes usually possess a good spectral selection and short response time.

In addition to conventional photodetectors based on p-n junction [12, 13], p-i-n photodiode [14], Schottky barrier [15], and metal-insulator-semiconductor [16] structures, recently, a new family of photoelectrochemical cell (PEC)-structured photodetectors has attracted tremendous academic interest and industrial attention due to its low-cost facile fabrication process and fast time response. Since the first report of a PEC self-powered ultraviolet photodetector (SPUV-PD) based on TiO_2_/water solid-liquid heterojunction by Lee and Hon [17], SPUV-PDs have been studied based on various wide bandgap semiconductor nanostructures, such as nanocrystalline TiO_2_ films [18], multilayer TiO_2_ nanorod-assembled cloth/nanorod arrays [19], and TiO_2_/SnO_2_ branched heterojunction [20] and ZnO nanoneedle arrays [21]. Vertical ZnO arrays have been considered as one of the most promising material for SPUV-PDs because of their high electron mobility and direct conduction path. However, compared with commercial UV detectors, the efficiency of ZnO nanorod-based SPUV-PDs is still low. This poor performance can be understood considering the defects commonly presented on the ZnO surface [22], which would lead to severe surface charge recombination and ultimately to a low efficiency of the ZnO nanorod-based devices. In order to further improve the performance of ZnO-based SPUV-PDs, more research work should be conducted to enhance the light-harvesting capability and reduce the carrier recombination. Forming a ZnO/ZnS heterostructure with a type-II band alignment may accelerate the charge separation, resulting in an improved photodetecting performance [23].

In the present work, a ZnO/ZnS core-shell nanorod array was developed by facile chemical solution methods and a PEC-type SPUV-PD was assembled using the ZnO/ZnS array as the active photoanode. Compared with SPUV-PDs based on bare ZnO nanorod arrays, this device shows improved photoresponsivity and higher sensitivity. This improved performance can be explained by the increased light-harvesting efficiency and accelerated separation of the photogenerated carriers caused by the ZnS coating layer. Considering the simple fabrication process, outstanding UV-detecting performance, and good stability, this kind of SPUV-PDs is a promising candidate for practical UV detector applications.

## Methods

### Sample Preparation

All chemicals used were analytical grade reagents without further purification. Both of the ZnO nanorods and ZnS nanoparticle shells were synthesized via facile chemical solution methods. The clean fluorine-doped tin oxide (FTO) substrates were spin-coated with 0.6 M zinc acetate in a mixed solution of ethanolamine and 2-methoxyethanol to acquire a seed layer of ZnO, followed by thermal treatment at 350 °C for 15 min. The seeded substrates were placed in a glass bottle with aqueous growth solution, which contain 20 mM zinc nitrate and 20 mM hexamethylenetetramine, and kept at 90 °C for 12 h. The growth process was repeated twice to obtain a desirable length of the ZnO nanorods. Subsequently, the substrates were taken out and rinsed with deionized water thoroughly and then annealed in air at 450 °C for 1 h to improve crystallinity and remove impurities. ZnS nanoparticle shells were prepared by a successive ionic layer adsorption and reaction (SILAR) method. In a typical SILAR cycle, a substrate pre-grown with ZnO nanorod arrays was dipped alternately into 0.1 M zinc nitrate aqueous solution for 1 min, rinsed with deionized water, then dipped into 0.1 M Na_2_S aqueous solution for another 1 min, and rinsed again with deionized water. This process was repeated for 2, 7, 10, and 15 cycles to achieve different thickness of ZnS nanoparticles.

### Device Assembling

The SPUV-PD was assembled in the same structure of a dye-sensitized solar cell as described in the previous paper [21]. In brief, the as-prepared ZnO or ZnO/ZnS nanorod array substrates were used as the active photoanode. The counter electrode was prepared by coating FTO glass with a thin layer of 8 mM H_2_PtCl_4_ ethanol solution, followed by heating at 400 °C for 20 min. The two electrodes were then sealed face to face with a 60-μm-thick sealing material. The internal space was filled with deionized water. The active area of the SPUV-PD was about 0.1 cm^2^.

### Characterization

X-ray diffraction (XRD; XD-3, PG Instruments Ltd., Beijing, China) was employed to identify the crystal structure of the samples. The morphologies of the samples were characterized by field-emission scanning electron microscopy (FESEM; Hitachi S-4800, Hitachi, Ltd., Chiyoda, Tokyo, Japan). The elemental composition of the samples was investigated using an energy-dispersive X-ray (EDX) spectrometer equipped on the FESEM. The absorption spectra were recorded on a UV-visible spectrophotometer (TU-1900, PG Instruments, Ltd., Beijing, China). The spectral responsivity characteristics and the typical current density-voltage (*J*-*V*) characteristics of the SPUV-PD based on ZnO or ZnO/ZnS nanorod arrays in the dark and under illumination were recorded using a programmable sourcemeter (2400, Keithley Instruments Inc., Cleveland, OH, USA). A 500-W xenon lamp (7ILX500, 7Star Optical Instruments Co., Beijing, China) equipped with a monochromator (7ISW30, 7Star Optical Instruments Co.) was used as the light source. For the time response measurement, a UV LED (NCSU033B (T), Nichia Co., Japan) with a wavelength of 365 nm was used as the light source and the photocurrent was measured using an electrochemical workstation (RST5200, Zhengzhou Shirusi Instrument Technology Co. Ltd, Zhengzhou, China).

## Results and Discussion

**Fig. 1:**
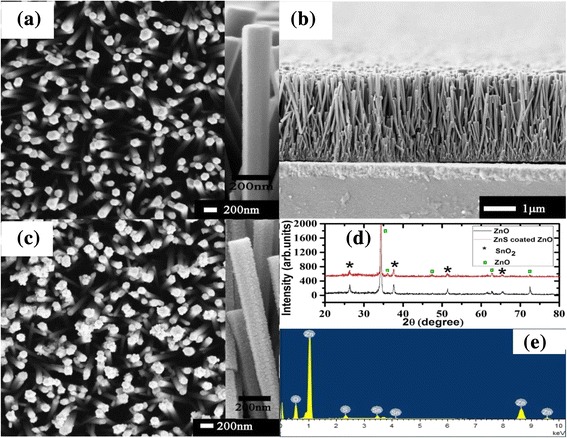
FESEM images and XRD patterns of ZnO and ZnO/ZnS core-shell nanorod arrays. **a** Top view FESEM image of ZnO nanorod array. Inset: high-magnification FESEM image of the ZnO nanorod. **b** Cross-sectional view of ZnO nanorod array. **c** Top view FESEM image of ZnO/ZnS core-shell nanorod array with seven SILAR cycles. Inset: high-magnification FESEM image of the ZnO/ZnS core-shell nanorod. **d** XRD patterns of ZnO nanorod arrays (*black line*) and ZnO/ZnS core-shell nanorod arrays (*red line*) grown on the FTO glass with seven SILAR cycles. **e** EDX data collected from the ZnO/ZnS core-shell nanorod arrays

The morphologies of the nanorod samples were examined by FESEM. Typical top and cross-section view FESEM images of ZnO nanorod arrays are displayed in Fig. 1a, b, which clearly show that the hexagonal ZnO nanorod arrays are grown vertically on the FTO glass. The diameter of hexagonal ZnO nanorods is approximately 100–200 nm. This nanorod array presents an easily accessed open structure for ZnS deposition. As shown in the cross-sectional view image, the ZnO nanorods on the FTO glass substrate have a length of about 4–5 μm. After coating ZnS with seven SILAR cycles, the surface of ZnO nanorod arrays was covered with nanoparticles. The magnified SEM image as an inset in Fig. 1c clearly reveals that ZnS nanoparticles were uniformly deposited on the surface of ZnO nanorod and form a thin shell resulting in a rough surface. This rough surface can be beneficial to light scattering. The crystal structure of ZnO and ZnO/ZnS core-shell nanorod arrays was identified by XRD, and all the results are shown in Fig. 1d. From the XRD data, it can be clearly seen that besides the SnO_2_ peaks from the FTO substrate, all other diffraction peaks could be attributed to the hexagonal wurtzite crystal structure of ZnO (JCPDS 36-1451). It is noteworthy that the (002) diffraction peak of ZnO was very high, which indicates that the ZnO nanorods are highly oriented along the *c*-axis direction with the growth axis perpendicular to the substrate surface. No diffraction peaks from ZnS can be observed because of its small particle size and poor crystalline quality. The elemental composition of the ZnO/ZnS core-shell nanorod arrays was further investigated using an EDX spectrometer. As shown in Fig. 1e, the Zn, O, and S peaks are from the core-shell nanorod arrays and the Sn peaks are from the FTO substrate. This result confirms the successful deposition of ZnS nanoparticle shell on the ZnO nanorod arrays.

**Fig. 2:**
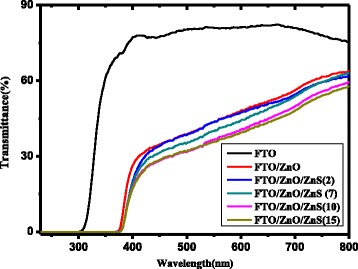
The UV-visible transmission spectrum of FTO glass, ZnO/FTO glass, and ZnO/ZnS/FTO glass with different ZnS SILAR cycles

Figure 2 shows the UV-visible transmission spectra of FTO glass, ZnO/FTO glass, and ZnS/ZnO/FTO glass photoanodes. The FTO glass shows an average transmittance of 80 % within the visible light region and a sharp absorption edge at about 310 nm. Compared with the bare FTO glass, the ZnO/FTO and ZnS/ZnO/FTO photoanodes show low transmittance (20 to 50 %) in the wavelength of the visible light region, which comes from the strong light scattering by the nanorod arrays. Moreover, the transmittance intensity in the visible range decreases with the increase of SILAR cycles. This can be attributed to the increased scattering effect induced by the rough ZnS shell. This enhanced light scattering will improve the light harvesting and results in a higher efficiency of the assembled photodetector. An obvious sharp absorption edge appears at about 385 nm, which can be attributed to the bandgap of wurtzite ZnO nanorod arrays. Taking both the transmission spectrum of the nanorod arrays and the FTO glass into consideration, we can achieve the conclusion that light with a wavelength between 310 and 385 nm can be effectively absorbed by these ZnO/FTO and ZnS/ZnO/FTO glass photoanodes and contribute to the photoresponse, which is very suitable for detecting UV-A (320–400 nm) light. This deduction will be further confirmed by the following photoresponsivity spectrum.

**Fig. 3:**
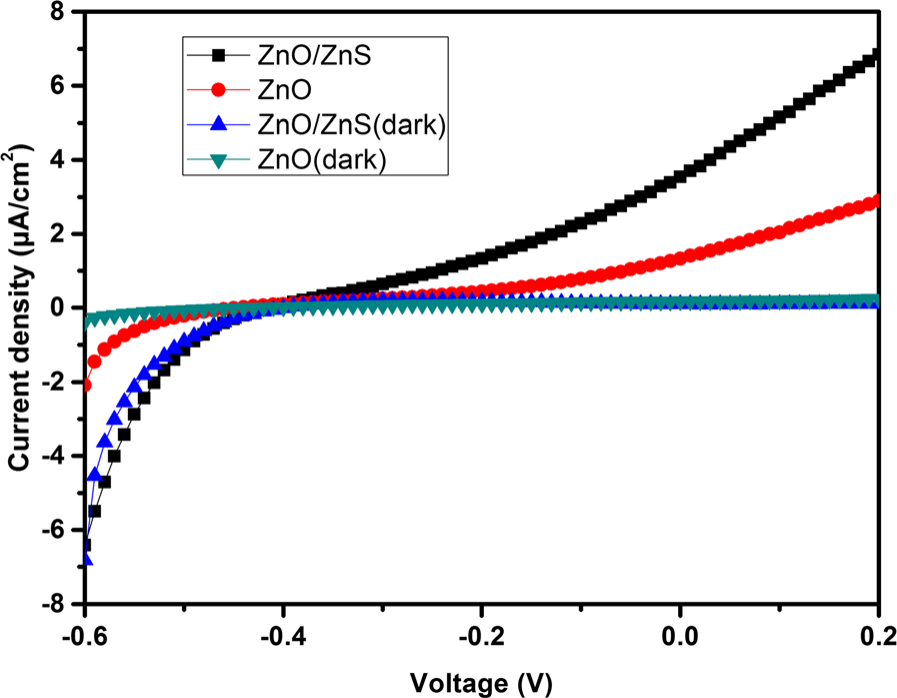
Current density-voltage curves of UV photodetectors based on bare ZnO and ZnO/ZnS core-shell nanorod arrays with seven ZnS SILAR cycles measured under dark and under 365-nm light illumination

Typical current density-voltage (*J*-*V*) curves of the self-powered photodetectors measured under dark and 365-nm light illumination are shown in Fig. 3. The dark *J-V* curve shows a forward turn-on voltage of about 0.4 V, which demonstrates a diode behavior with significant rectification characteristics. Under the illumination of 0.0812 mW/cm^2^ UV light (*λ* = 365 nm), the ZnO/ZnS (with seven SILAR cycles) core-shell nanorod array-based SPUV-PD shows a much better photovoltaic performance compared with that based on bare ZnO nanorods, yielding an open-circuit voltage of 0.41 V and a short-circuit current density of 3.54 μA/cm^2^, while the ZnO nanorod-based photodetector only has a short-circuit current density of 1.37 μA/cm^2^. This remarkable increase should be attributed to the deposition of the ZnS nanoparticles on the ZnO nanorods. The inherent built-in potential arises from the ZnO/ZnS-water interface, acts as a driving force to separate the photogenerated electron-hole pairs, and increases the photocurrent. Therefore, this device can operate at photovoltaic mode without any external bias.

**Fig. 4:**
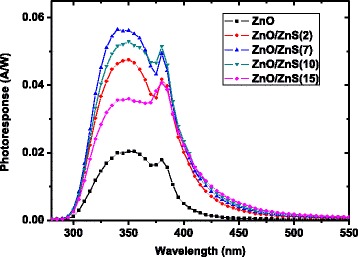
Spectral responsivity characteristics of UV photodetectors based on ZnO nanorods and ZnO/ZnS core-shell nanorod arrays with different SILAR cycles under 0-V bias

The spectral photoresponsivity results of SPUV-PD based on the bare ZnO nanorods and the ZnO/ZnS core-shell nanorod arrays with different ZnS SILAR cycles at 0-V bias are displayed in Fig. 4. The incident light wavelength ranges from 280 to 550 nm. The maximum photoresponsivity is only 0.02 A/W for the SPUV-PD based on bare ZnO nanorods. After coating ZnS with two SILAR cycles, the photoresponsivity is more than 0.03 A/W in the wavelength region from 320 to 390 nm. These results suggest that the ZnO/ZnS core-shell nanorod array is benefited to improve the photoresponsivity of SPUV-PDs. The best photoresponsivity of 0.056 A/W is obtained for the sample with ZnS seven SILAR cycles at 340 nm. It is noteworthy that the photoresponsivity is more than 0.04 A/W in the range of 320 to 400 nm. When ZnS SILAR cycles further increased, more ZnS nanoparticles are deposited onto the ZnO nanorods, and the photoresponsivity of ZnO/ZnS array UV photodetectors decreased. This demonstrates that a thick ZnS nanoparticle layer may hinder the hole transport and enhance the recombination reaction of the photon-generated carrier. In addition to the high sensitivity, the photodetector also exhibits an excellent wavelength selectivity in the spectral range between 310 and 400 nm, which is suitable for the UV-A range. The responsivity drops fast to nearly zero on the short-wavelength side because of the absorption edge of the FTO glass, the photoresponsivity also decreases rapidly as the wavelength is longer than 450 nm because of the low absorption for photons with energies smaller than the bandgap of ZnO. It should be noted that a weak photoresponse appears above the cutoff response wavelength of ZnO up to 500 nm. This phenomenon is caused by the slight light absorption of the defects in ZnS nano-sized particles and can be reduced by improving the crystalline quality of ZnS.

**Fig. 5:**
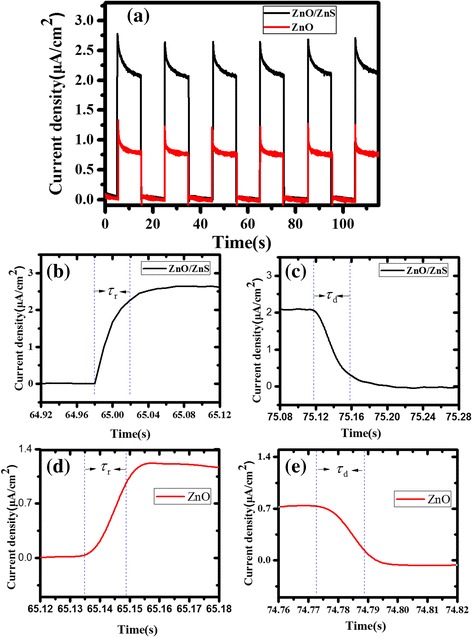
The real-time photocurrent response of the ZnO/ZnS core-shell nanorod array-based UV photodetector with seven SILAR cycles. **a** Photocurrent response under on/off UV light radiation with the illumination wavelength of 365 nm. **b** Enlarged rising and **c** decaying edges of the photocurrent response under 365-nm UV light illumination. **d** Enlarged rising and **e** decaying edges of the ZnO nanorod array-based photodetectors

Real-time photocurrent response is a key parameter for a photodetector. The photocurrent time response of the self-powered UV detectors was recorded at a zero bias under on/off switching radiation of 365-nm UV light with an on/off interval of 10 s. As shown in Fig. 5a, the photocurrent was observed to be consistent and repeatable with no degenerate effect during six repeat cycles. A fast photoresponse can be seen clearly by the enlarged rising and decaying edges of the photocurrent response shown in Fig. 5b–e. Both the decaying and rising times (defined as the time required for the photocurrent to increase from minimum to 90 % and drop from maximum to 10 % of the maximum photocurrent) of the ZnO nanorod array-based photodetector are less than 0.02 s, while the ZnO/ZnS core-shell array-based photodetector exhibits a longer response time of about 0.04 s. As the ZnS is deposited at room temperature, many defects may form in the ZnS particles. These defects can trap charges and slow the response time.

**Fig. 6:**
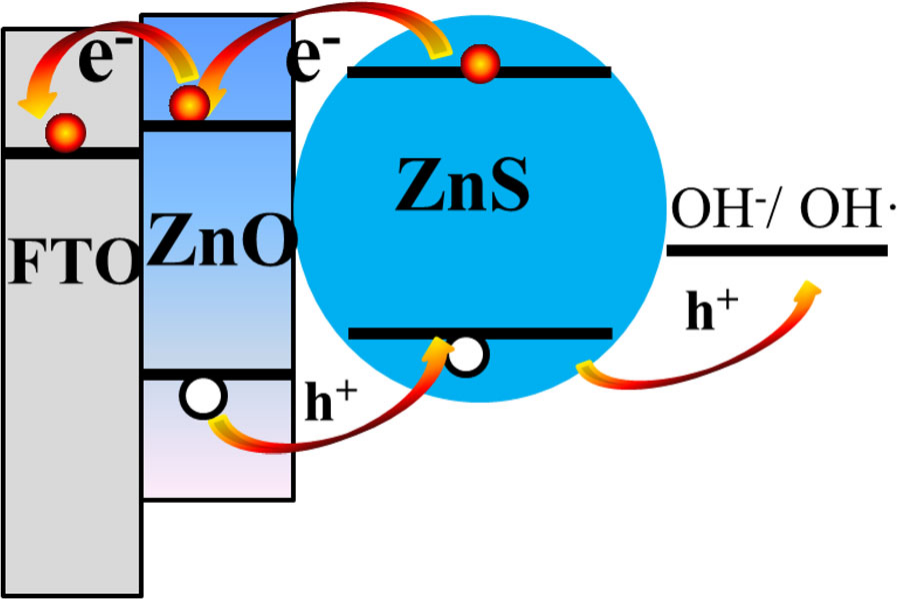
Schematic energy band diagram of ZnO/ZnS core-shell nanorod arrays and the charge-transfer processes

The self-powered photodetector based on the ZnO/ZnS core-shell nanorod array photoanode shows an improved photoresponsivity compared with that based on bare ZnO nanorod arrays. Although the reason for this remarkable improvement is not quite clear, we propose that it may be explained by the following two aspects: (i) ZnS nanoparticles coated on a ZnO nanorod array structure may increase the UV light scattering, resulting in an enhancement of the photon-harvesting efficiency. (ii) The band energy alignment of the ZnO/ZnS core-shell nanorod array is favorable to the spatial separation of the photogenerated carriers and the charge transfer. The values of the lower edge of the conduction band for ZnS and ZnO were around −3.46 and −4.19 eV, respectively, whereas the values of the upper edge of the valence band were around −7.06 and −7.39 eV, respectively [24]. Taking both bandgaps of ZnO and ZnS into consideration, a type-II band edge structure at the ZnO/ZnS interface as shown in Fig. 6 could be formed. As a result, when both the ZnO and ZnS are photoexcited under UV light illumination, the electrons will move to the ZnO side and holes will move to the ZnS side due to the staggered bandgap alignment, facilitating the formation of a charge transfer state and the spatial separation of the photogenerated carriers within the ZnO/ZnS nanorod arrays. The spatial separation of the photogenerated carriers can decrease the recombination of the electron-hole pairs and therefore significantly increase the photocurrent and photoresponsivity of the ZnO/ZnS core-shell nanorod array-based SPUV-PD [25].

Our results indicate that ZnO/ZnS core-shell nanorod arrays can be used as an active photoanode for high-performance photoelectrochemical cell-type self-powered ultraviolet photodetectors, whereas the liquid electrolyte in devices may result in some practical limitations of sealing and long-term stability. Therefore, solid-state electrolytes should be developed for this structure of SPUV-PD to realize its commercial application.

## Conclusions

In summary, vertical ZnO nanorod arrays were uniformly grown on FTO glass by a hydrothermal method. ZnS nanoparticle shells were deposited onto ZnO nanorods by a SILAR method. A self-powered, low-cost, photoelectrochemical cell-structured UV light photodetector was assembled using the ZnO/ZnS core-shell nanorod arrays as the photoanode. The experimental results show that, compared with a detector based on bare ZnO nanorod arrays, the ZnO/ZnS photoanode demonstrates a slightly slower response time but a much higher photosensitivity. The higher efficiency of the ZnO/ZnS core-shell nanostructures can be attributed to the synergistic effect of greatly enhanced light harvesting, reduced charge recombination, and accelerated charge transfer. All these results indicate the great potential of the ZnS/ZnO nanorod array-based SPUV-PD for high-sensitivity and high-speed UV light detecting.

## References

[1] Jin YZ, Wang JP, Sun BQ, Blakesley JC, Greenham NC (2008). Solution-processed ultraviolet photodetectors based on colloidal ZnO nanoparticles. Nano Lett.

[2] Zhou JY, Chen LL, Wang YQ, He YM, Pan XJ, Xie RQ (2016). An overview on emerging photoelectrochemical self-powered ultraviolet photodetectors. Nanoscale.

[3] Hu LF, Yan J, Liao MY, Wu LM, Fang XS (2011). Ultrahigh external quantum efficiency from thin SnO_2_ nanowire ultraviolet photodetectors. Small.

[4] Hsu CL, Chang SJ (2014). Doped ZnO 1D nanostructures: synthesis, properties, and photodetector application. Small.

[5] Tsai SY, Hon MH, Lu YM (2011). Fabrication of transparent p-NiO/n-ZnO heterojunction devices for ultraviolet photodetectors. Solid State Electron.

[6] Bai S, Wu WW, Qin Y, Cui NY, Bayerl DJ, Wang XD (2011). High-performance integrated ZnO nanowire UV sensors on rigid and flexible substrates. Adv Funct Mater.

[7] Tsai T, Chang SJ, Weng WY, Hsu CL, Wang SH, Chiu CJ, Hsueh TJ, Chang SP (2012). A visible-blind TiO_2_ nanowire photodetector. J Electrochem Soci.

[8] Liu ML, Weng TM, Chen JY, Chen YF (2012). Ultrahigh-gain single SnO_2_ nanowire photodetectors made with ferromagnetic nickel electrodes. NPG Asia Mater.

[9] Liu H, Zhang ZM, Liao MY, Ma RZ, Xu FF, Fang XS (2014). New UV-A photodetector based on individual potassium niobate nanowires with high performance. Adv Optical Mater.

[10] Kind H, Yan HQ, Messer B, Law M, Yang PD (2002). Nanowire ultraviolet photodetectors and optical switches. Adv Mater.

[11] Fang XS, Bando Y, Liao MY, Gautam UK, Zhi CY, Dierre B, Liu BD, Zhai TY, Sekiguchi T, Koide Y, Golberd D (2009). Single-crystalline ZnS nanobelts as ultraviolet-light sensors. Adv Mater.

[12] Shen YW, Xiaoqin Yan XQ, Bai ZM, Zheng X, Sun YH, Liu YC, Lin P, Xiang Chen X, Zhang Y (2015). A self-powered ultraviolet photodetector based on solution-processed p-NiO/n-ZnO nanorod array heterojunction. RSC Adv.

[13] Shi LL, Wang F, Li BH, Chen X, Yao B, Zhao DX, Shen DZ (2014). A highly efficient UV photodetector based on a ZnO microwire p–n homojunction. J Mater Chem C.

[14] Xu GY, Salvador A, Kim W, Fan Z, Lu C, Tang H, Morkoc H (1997). High speed, low noise ultraviolet photodetectors based on GaN pin and AlGaN (p)-GaN (i)-GaN (n) structures. Appl Phys Lett.

[15] Liang S, Sheng H, Liu Y, Huo Z, Lu Y, Shen H (2001). ZnO Schottky ultraviolet photodetectors. J Cryst Growth.

[16] Ali GM, Chakrabarti P (2010). Effect of thermal treatment on the performance of ZnO based metal-insulator-semiconductor ultraviolet photodetectors. Appl Phys Lett.

[17] Lee WJ, Hon MH (2011). An ultraviolet photo-detector based on TiO_2_/water solid-liquid heterojunction. Appl Phys Lett.

[18] Li XD, Gao CT, Duan HG, Lu BG, Pan XJ, Xie EQ (2012). Nanocrystalline TiO_2_ film based photoelectrochemical cell as self-powered UV-photodetector. Nano Energy.

[19] Wang ZR, Ran SH, Liu B, Chen D, Shen GZ (2012). Multilayer TiO_2_ nanorod cloth/nanorod array electrode for dye-sensitized solar cells and self-powered UV detectors. Nanoscale.

[20] Li XD, Gao CT, Duan HG, Lu BA, Wang YQ, Chen LL, Zhang ZX, Pan XJ, Xie EQ (2013). High-performance photoelectrochemical-type self-powered UV photodetector using epitaxial TiO_2_/SnO_2_ branched heterojunction nanostructure. Small.

[21] Li QH, Wei L, Xie YR, Zhang K, Liu L, Zhu DP, Yan SH, Jiao J, Liu GL, Mei LM (2013). ZnO nanoneedle/H_2_O solid-liquid heterojunction-based self-powered ultraviolet detector. Nanoscale Res Lett.

[22] Qin LQ, Shing C, Sawyer S, Dutta PS (2011). Enhanced ultraviolet sensitivity of zinc oxide nanoparticle photoconductors by surface passivation. Opt Mater.

[23] Huang X, Wang M, Willinger MG, Shao LD, Su DS, Meng XM (2012). Assembly of three-dimensional hetero-epitaxial ZnO/ZnS core/shell nanorod and single crystalline hollow ZnS nanotube arrays. ACS Nano.

[24] Xu Y, Schoonen MAA (2000). The absolute energy positions of conduction and valence bands of selected semiconducting minerals. Am Mineral.

[25] Hu LF, Yan J, Liao MY, Xiang HJ, Gong XG, Zhang LD, Fang XS (2012). An optimized ultraviolet-A light photodetector with wide-range photoresponse based on ZnS/ZnO biaxial nanobelt. Adv Mater.

